# The Association Between Monocyte Subsets and Cardiometabolic Disorders/Cardiovascular Disease: A Systematic Review and Meta-Analysis

**DOI:** 10.3389/fcvm.2021.640124

**Published:** 2021-02-17

**Authors:** Ester S. Oh, Muzi Na, Connie J. Rogers

**Affiliations:** ^1^Department of Nutritional Sciences, The Pennsylvania State University, University Park, PA, United States; ^2^Center for Molecular Immunology and Infectious Disease, Huck Institutes of the Life Sciences, The Pennsylvania State University, University Park, PA, United States

**Keywords:** monocytes, inflammation, cardiometabolic disorder, cardiovascular disease, systematic review

## Abstract

**Background:** Monocyte subsets in humans, i.e., classical (CM), intermediate (IM), and non-classical monocytes (NCM), are thought to differentially contribute to the pathogenesis of atherosclerosis, the leading cause of cardiovascular disease (CVD). However, the association between monocyte subsets and cardiometabolic disorders and CVD is not well-understood. Thus, the aim of the current systematic review and meta-analysis was to evaluate recent findings from clinical studies that examined the association between the distribution of monocyte subsets in subjects with cardiometabolic disorders and CVD compared to healthy controls.

**Methods:** Articles were systematically searched in CINAHL, PubMed and Cochrane Library. Articles were independently screened and selected by two reviewers. Studies that reported the percentage of each monocyte subset were included in the systematic review and meta-analysis. For the meta-analysis, a random-effects model was used to generate pooled standardized mean differences (SMD) between subjects with cardiometabolic disorders and healthy controls.

**Results:** A total of 1,693 articles were screened and 27 studies were selected for qualitative analyses. Among them, six studies were included in the meta-analysis. In total, sample size ranged from 22 to 135 and mean or median age from 22 to 70 years old. We found studies that reported higher percentage and number of IM and/or NCM in subjects with cardiometabolic disorders (9 out of 13 studies) and in subjects with CVD (11 out of 15 studies) compared to healthy controls. In the meta-analysis, the percentage of CM was lower [SMD = −1.21; 95% CI (−1.92, −0.50); *P* = 0.0009; *I*^2^ = 91%] and the percentage of IM [SMD = 0.56; 95% CI (0.23, 0.88); *P* = 0.0008; *I*^2^ = 65%] and NCM [SMD = 1.39; 95% CI (0.59, 2.19); *P* = 0.0007; *I*^2^ = 93%] were higher in subjects with cardiometabolic disorders compared to healthy controls.

**Conclusions:** Individuals with cardiometabolic disorders and CVD may have a higher percentage of IM and NCM than healthy controls. Future studies are needed to evaluate the cause and biological significance of this potential altered distribution of monocyte subsets.

## Introduction

In 2015, there were an estimated 422.7 million cases of cardiovascular disease (CVD) worldwide resulting in 17.9 million CVD deaths (1). Among the different types of CVD, coronary heart disease was the leading cause of morbidity globally, followed by hemorrhagic and ischemic stroke, and hypertension (2, 3). Atherosclerosis is the dominant cause of coronary heart disease and stroke (4), which are linked to myocardial infarction and heart failure (5, 6). Risk factors for CVD such as obesity, hypercholesterolemia, and type 2 diabetes, have proatherogenic effects by accelerating endothelial and vascular smooth muscle dysfunction, vascular infiltration of low-density lipoprotein (LDL), and the production of oxidized LDL in the subendothelial space (7, 8). Moreover, many studies have demonstrated a strong association between inflammation and the risk of atherosclerosis and future cardiovascular events (9–11). Unresolved and/or dysregulated inflammation triggered by the aforementioned cardiometabolic disorders participates in all stages of atherosclerosis via activation of the innate immune system (12), as well as the expression of adhesion molecules on the arterial surface to induce leukocyte adherence and migration into the intima (13). During the development and progression of atherosclerosis, monocytes play a central role in the dynamic interplay between T lymphocytes, smooth muscle cells, and platelets in the vascular endothelium (14).

Monocytes are one of the key innate immune cells that circulate in the blood, recognize and attack pathogens, and differentiate into monocyte-derived macrophages (15). Human monocytes express varying amounts of the lipopolysaccharide (LPS) receptor (CD14) and the low-affinity Fc receptor III for IgG (CD16) on their cell surface, and are classified as classical (CD14^++^CD16^−^; CM), intermediate (CD14^++^CD16^+^; IM) and non-classical (CD14^+^CD16^++^; NCM) monocytes (16). Each monocyte subset displays distinct phenotypic and functional properties which is thought to differentially orchestrate key aspects of the inflammatory response (16). CM are reported to have high phagocytic activity featured by the expression of genes that regulate phagocytosis (17). Upon stimulation with LPS, CM primarily release proinflammatory cytokines, including IL-1β, IL-6, MCP-1, and TNF-α (18, 19). Moreover, CM have the greatest trans-endothelial migration capacity among the three monocyte subsets (20, 21). IM are in a transitional state from CM to NCM and share phenotypic and functional properties of both subsets (16). IM also are reported to contribute to the inflammatory response via the production of proinflammatory cytokines, such as IL-6 and TNF-α, upon LPS stimulation (18, 19). IM are attracted to atherosclerotic lesions via their expression of C-C chemokine receptor type 5 (CCR5) and are thought to play a role in the pathogenesis of atherosclerosis (17, 22). NCM patrol the vascular endothelium for the presence of damaged cells and may have a surveillance role (23–25).

Several recent observational studies highlight the importance of characterizing monocyte subsets and hypothesize that monocyte subsets may play a differential role in the risk and progression of atherosclerosis and CVD (26, 27). However, the relationship between the distribution of human monocyte subsets and the risk of cardiometabolic disorders and/or CVD has not been systematically evaluated. Therefore, the aim of the current review was to summarize and evaluate findings reported in clinical studies that examined changes in the distribution of monocyte subsets in subjects with cardiometabolic disorders (obesity, metabolic syndrome, hypercholesterolemia, and type 2 diabetes) and CVD (atherosclerosis, coronary artery/heart disease, acute/chronic heart failure, acute myocardial infarction, and acute coronary syndrome) compared to healthy controls to determine if an altered distribution of monocyte subsets is associated with the presence of cardiometabolic disorders and/or CVD.

## Methods

### Protocol Registration

This systematic review was conducted based on the Preferred Reporting Items for Systematic Reviews and Meta-Analysis (PRISMA) statement (28) (Supplementary Table 1), and was registered in PROSPERO (CRD42020155794) prior to screening and selecting articles.

### Search Strategy

A search strategy was developed in collaboration with a health science librarian at the Pennsylvania State University–University Park campus. The search strategy included two groups of terms to reflect the key concepts of cardiometabolic disorders and CVD (population) and monocyte subsets (outcome) in humans in order to compare the distribution of monocyte subsets between healthy subjects and those with cardiometabolic disorders and/or CVD. Detailed search terms, filters and number of results are provided in Supplementary Table 2. The search was conducted using three databases including Cumulative Index for Nursing and Allied Health (CINAHL), Cochrane Central Register of Controlled Trials, and PubMed, and article records were managed in EndNote X8 (Clarivate Analytics). This search was conducted in October 2020.

### Inclusion and Exclusion Criteria

Inclusion and exclusion criteria were established before study selection (Table 1). Eligible studies included adults ≥18 years of age. Eligible studies were cross-sectional, cohort, within-subject design studies and randomized-controlled trials (data from baseline) that compared the distribution of monocyte subsets between healthy subjects and those with cardiometabolic disorders and/or CVD. Studies were excluded if they lacked a healthy control group. Animal studies, reviews, abstracts, editorials, commentaries and book chapters were excluded.

**Table 1:** Inclusion and exclusion criteria.

**Components**	**Inclusion criteria**	**Exclusion criteria**
Date range	December 1970–October 2020	-
Language range	English only	-
Population	Adults (≥18 years old)	-
Intervention	No intervention	-
Control	-	No healthy controls
Outcome	Changes in the distribution, phenotype and function of monocyte subsets in humans	-
Study design	Study designs that were eligible for this review includes: cross-sectional, cohort, within-subject design studies and randomized-controlled trials (baseline data) that compare the distribution of monocyte subsets between healthy participants and those with cardiovascular risk/diseases	Animal studies
Publication format	-	Reviews, conference abstracts, editorials, commentaries, and book chapters

### Study Selection and Data Extraction

ESO and CJR independently screened articles by titles and abstracts based on inclusion and exclusion criteria. Articles considered potentially relevant by each reviewer were included for full-text review.

Data were extracted by ESO into a standardized spreadsheet and verified by CJR. Extracted data include: first author, publication year, study design, number of participants, characteristics of study population (type of cardiometabolic disorders and CVD), sex and mean/median age of participants. The types of cardiometabolic disorders (overweight and obesity, metabolic syndrome, hypercholesterolemia and type 2 diabetes) and CVD (atherosclerosis, coronary artery disease, coronary heart disease, chronic heart failure, acute heart failure, acute myocardial infarction, and acute coronary syndrome) were chosen because of their association with elevated systemic inflammatory mediators (9, 10, 12, 13). The differences in the distribution of monocyte subsets in participants with cardiometabolic disorders or CVD compared to healthy controls were extracted for each included study. For studies that had multiple groups of subjects with different cardiometabolic disorders or CVD compared to healthy controls, the data were extracted for each condition separately.

From articles that reported the percentage of monocyte subsets, data (mean/median, SD/range and *P*-value) were extracted and used for meta-analysis. From articles that reported correlation coefficients between each monocyte subset and clinical parameters including age, BMI, waist circumference, fat mass %, fasting blood glucose (FBG), HbA1c, fasting insulin, Homeostatic Model Assessment of Insulin Resistance (HOMA-IR), triglycerides (TG), total cholesterol (TC), LDL-cholesterol (LDL-C), high-density lipoprotein cholesterol (HDL-C), very low-density lipoprotein (VLDL), and C-reactive protein (CRP), data (correlation coefficients) were extracted and used for meta-analysis of correlation coefficients.

### Quality Assessment

The NIH National Heart, Lung, and Blood Institute (NHLBI) Quality Assessment Tool for Observational Cohort and Cross-sectional Studies was used to assess the quality of observational studies (29). The quality was rated as excellent (12–14 points), good (8–11 points), fair (4–7 points), and poor (0–3 points). The Risk Of Bias In Non-randomized Studies of Interventions (ROBINS-I) tool was used to assess the quality of non-randomized studies (30). ESO and CJR independently assessed the quality of included studies. Any discrepancies were resolved by discussion.

### Statistical Analyses of Meta-Analysis

The mean and SD of the percentage of each monocyte subset from the group with cardiometabolic disorders and the healthy control group were pooled into standardized mean difference and 95% confidence interval (CI) to evaluate the association between cardiometabolic disorders and the distribution of monocyte subsets. The mean and SD were extracted from articles, and a standard formula was used to derive the mean and SD if data were reported in a different statistical measures (31). For studies that had multiple groups of subjects with different cardiometabolic disorders and/or CVD compared to healthy controls, the data were included separately in the analysis. A meta-analysis evaluating the association between the distribution of monocyte subsets and cardiometabolic disorders was performed with Cochrane Review Manager (RevMan) version 5.3 (32). A random-effects model was used to generate pooled effect estimates allowing for differences of observed outcomes (distribution of each monocyte subset) across studies. The among-study variance (tau-squared, τ^2^), chi-squared (χ^2^) test and Higgin's *I*^2^ statistics were used to test for statistical heterogeneity. Statistical significance was accepted at *P* < 0.05. Funnel plots were generated with RevMan version 5.3 (32) to examine publication bias of studies included in the meta-analysis (Supplementary Figure 1).

A meta-analysis of correlation coefficients between monocyte subsets and clinical parameters was performed using MedCalc Statistical Software version 19.1.7 (MedCalc Software, Ostend, Belgium; https://www.medcalc.org). A random-effects model was used to provide a more conservative estimate of the correlation between monocyte subsets and clinical parameters. Each study was weighted according to the number of participants included. Higgin's *I*^2^ statistics were used to test for statistical heterogeneity. Statistical significance was accepted at *P* < 0.05.

## Results

### Study Selection

**Figure 1:**
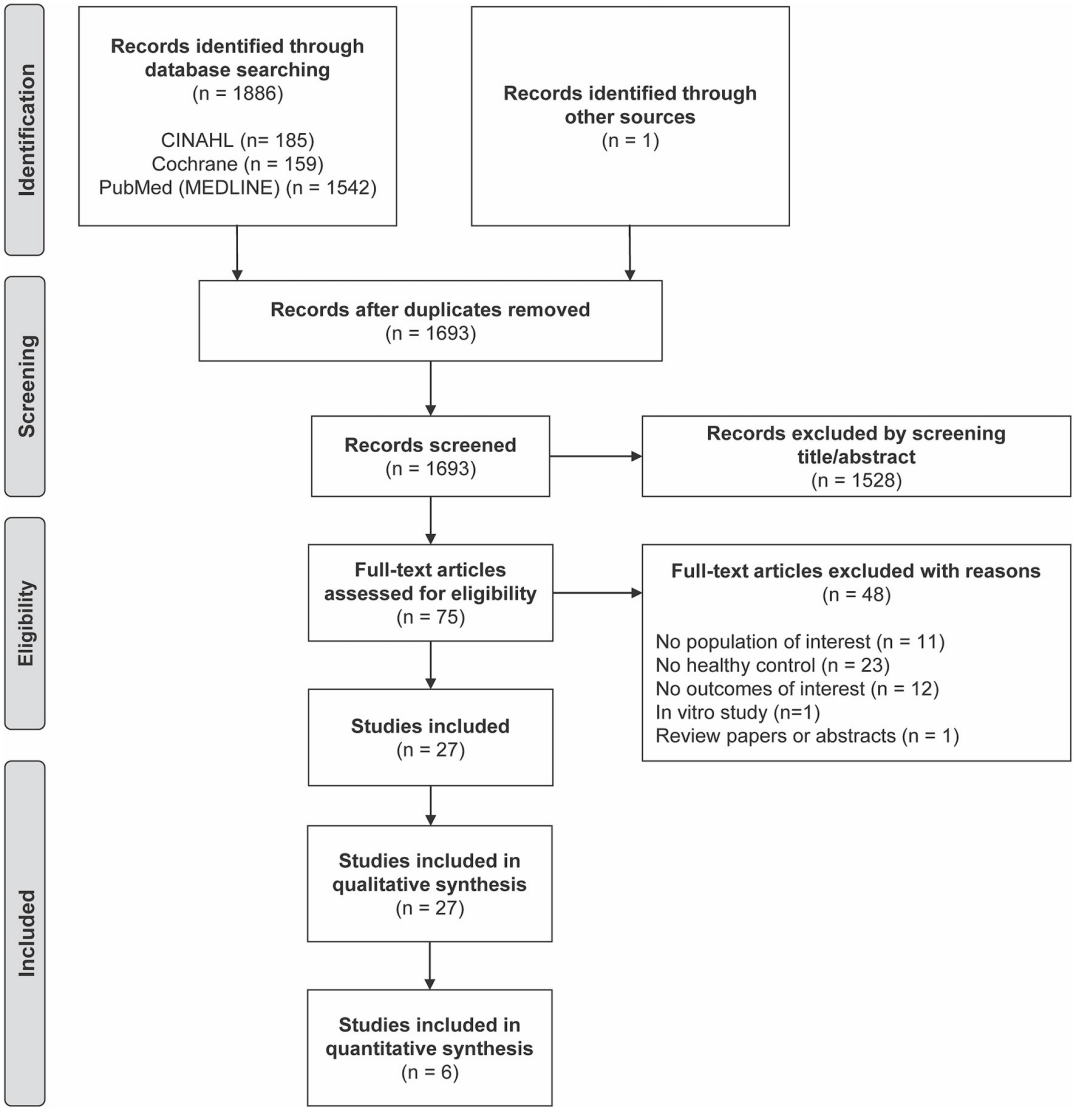
Preferred Reporting Items for Systematic Reviews and Meta-Analyses (PRISMA) flow diagram of included studies.

A total of 1,887 articles were identified through database searching, leaving 1,693 articles for title and abstract screening after duplicates were removed (Figure 1). We excluded 1,528 articles according to the inclusion and exclusion criteria (Table 1), leaving 75 articles eligible for a full-text review. Additional 48 publications were removed because they did not meet the criteria, as detailed in Figure 1. As a result, 27 studies were included in the qualitative analyses, which examined the association between cardiometabolic disorders and CVD and the distribution of monocyte subsets. Among the articles, six studies were included in the meta-analysis to evaluate the association between cardiometabolic disorders and the distribution of each monocyte subset.

### Characteristics of Included Studies

This review included 26 cross-sectional studies and one within-subject design study that investigated the association between the cardiometabolic disorders and/or CVD and the distribution of monocyte subsets (Table 2). The types of cardiometabolic disorders included are overweight (*n* = 2), obesity (*n* = 6), metabolic syndrome (*n* = 2), hypercholesterolemia (*n* = 2), and type 2 diabetes (*n* = 4). The types of CVD included are atherosclerosis (*n* = 3), coronary artery disease (*n* = 6), coronary heart disease (*n* = 1), chronic heart failure (*n* = 2), acute heart failure (*n* = 1), acute myocardial infarction (*n* = 3), and acute coronary syndrome (*n* = 1). The sample sizes ranged from 22 to 135 and the mean/median age from 22 to 70 years old.

**Table 2:** Included studies examining the association between the distribution of monocyte subsets and cardiometabolic disorders.

**Reference**	**Study design^a^**	**Subjects**			**Outcomes**				
		**Clinical conditions**	***N* (Male %)**	**Age^b^**	**Distribution of monocyte subsets^c^**				**Other outcomes related to monocytes^d^**
					**Unit**	**CM**	**IM**	**NCM**	
**Overweight**									
Friedrich et al. (33)	CSS	Healthy Overweight	27 (7) 23 (9)	43 46	Number	↑	↑	~	– The number of CM and IM was positively correlated with BMI, fat mass, waist circumference, triglycerides and CRP, and negatively correlated with HDL cholesterol.
Poitou et al. (34)	CSS	Healthy Overweight	32 (22) 39 (21)	34 43	% Number	↓ ↓	~ ~	~ ~	– The percentage of IM was positively correlated with BMI, fat mass, glucose, insulin, and HOMA-IR.
**Obesity**									
Boersema et al. (35)	CSS	Healthy Obesity	20 (10) 20 (10)	42 41	%	~	~	~	-
Christou et al. (36)	CSS	Healthy Obesity	25 (40) 58 (47)	38 46	% Number	↓ ~	↑ ↑	~ ↑	– In subjects with obesity, the number of IM was positively correlated with total cholesterol, triglycerides, non-HDL cholesterol, ApoB, systolic blood pressure, and diastolic blood pressure. – In subjects with obesity, the number of NCM was positively correlated with body weight, insulin, HOMA-IR, total cholesterol, LDL cholesterol, and non-HDL cholesterol.
Devêvre et al. (37)	CSS	Healthy Obesity	26 (8) 40 (10)	37 41	% Number	~ ↓	↑ ↑	↑ ↑	– The percentage of monocytes in PBMC was higher in subjects with obesity than healthy controls. – There was a higher expression of CCR2, CCR5 on CM and IM and a higher expression of CX3CR1 on all three monocyte subsets in subjects with obesity compared to healthy controls. – There was a higher TLR4-stimulated secretion of IL-1β and CCL5 in CM and IM and a higher TLR8-stimulated secretion of TNF-α and IL-10 in CM in subjects with obesity compared to healthy controls. – In all three monocyte subsets, the expression of CCR5 was positively correlated with triglycerides and glucose, and the expression of CX3CR1 and CCR2 was negatively correlated with HDL cholesterol.
Friedrich et al. (33)	CSS	Healthy	27 (7)	43	Number	↑	↑	~	(see above)
		Obesity (45% T2D)	60 (17)	46					
Krinninger et al. (38)	CSS	Healthy Obesity	16 (0) 14 (0)	35 36	%	↓	↑	ND	– The migration of monocytes was higher in women with obesity compared to healthy women. – A higher expression of CCR2 and CCR5 on CM and IM was observed in women with obesity compared to healthy women, which may contribute to a stronger adhesion and migration properties of CM and IM in obesity.
Poitou et al. (34)	CSS	Healthy Obesity	32 (22) 67 (15)	34 38	% Number	↓ ↓	↑ ↑	↑ ↑	(see above)
**Metabolic syndrome (MS)**									
Grün et al. (39)	CSS	Healthy MS	42 (60) 44 (64)	49 48	%	↓	~	↑	– In healthy subjects, the percentage of CM was negatively correlated with BMI and body fat, and the percentage of NCM was positively correlated with BMI, waist circumference and body fat. – In subjects with MS, the percentage of CM was positively correlated with HDL cholesterol, while the percentage of NCM was negatively correlated with HDL cholesterol.
Khan et al. (40)	WSD	Healthy MS	11 (82) 11 (82)	43 60	Number	~	~	↑	– The percentage of foamy monocytes was higher in subjects with MS compared to healthy controls. – The percentage of foamy monocytes was positively correlated with circulating triglycerides.
**Hypercholesterolemia (HC)**									
Jaipersad et al. (41)	CSS	Healthy HC	40 (43) 40 (43)	67 67	Number	~	~	~	– In subjects with HC, there was a higher IL-6R, CD49d, and CXCR4 expression on CM, a higher IL6R and CXCR4 expression on IM, and a higher CXCR4 expression on NCM compared to healthy controls.
Nielsen et al. (42)	CSS	Healthy Familial HC	23 (35) 30 (40)	47 46	%	~	↑	~	– The expression of CD36, which involves in the uptake of oxidized LDL cholesterol, on NCM was higher in subjects with HC compared to healthy controls.
**Type 2 diabetes (T2B)**									
Poitou et al. (34)	CSS	Healthy	32 (22)	34	%	↓	↑	↑	(see above)
		T2D and Obesity	38 (32)	50	Number	↓	↑	↑	
Von Scholten et al. (43)	CSS	Healthy T2D	16 (63) 37 (70)	61 62	Number	~	~	↓	– There was a higher MFI of CD11c, which involves in monocyte adherence and migration into atherosclerotic plaques, in patients with T2D compared to healthy controls.
Zaharieva et al. (44)	CSS	Healthy T2D	7 (14) 28 (36)	52 54	%	↓	~	↑	– The percentage of IM expressing CD163 was higher in patients with type 2 diabetes compared to healthy controls. – CD163 expressing IM were positively associated with waist circumference and glucose.
Valtierra-Alvarado et al. (45)	CSS	Healthy T2D	27 (ND) 28 (ND)	43 47	%	↓	~	~	– The percentage CM was negatively correlated with BMI, HbA1c, and glucose. – The percentage of NCM was positively correlated with BMI, HbA1c, and glucose.

a *Study design: CSS; cross-sectional study; WSD, within-subject design*.

b *Age: Age is presented as in the original article (mean or median)*.

c *Distribution of monocyte subsets: Unit number, the absolute counts of monocyte subsets (cells/μl); Unit %, the percent distribution of monocyte subsets; ↑/↓ monocyte subsets in subjects with cardiometabolic disorder are significantly higher/lower compared to healthy controls*.

d *Other outcomes related to monocytes: Outcomes with significant difference are presented*.

*ApoB, apolipoprotein B; BMI, body mass index; CCL, C-C Motif Chemokine Ligand; CCR, C-C Motif Chemokine Receptor; CM, classical monocytes; CRP, C-reactive protein; CXCR4, C-X-C chemokine receptor type 4; CX3CR1, CX3C chemokine receptor 1; HbA1c, Hemoglobin A1c; HDL, high-density lipoprotein; HOMA-IR, Homeostatic Model Assessment of Insulin Resistance, IL, interleukin; IL6R, interleukin 6 receptor; IM, intermediate monocytes; LDL, low-density lipoprotein; MFI, mean (median) fluorescence intensity; NCM, non-classical monocytes; PBMC; peripheral blood mononuclear cells; TLR, Toll-like receptor; TNF, Tumor Necrosis Factor*.

### Quality of Included Studies

Based on NHLBI Quality Assessment Tool for Observational Cohort and Cross-sectional Studies, 31% of cross-sectional studies were in good quality and 65% in fair quality, and 4% in poor quality (Supplementary Table 3). According to ROBMINS-I, the overall risk of a non-randomized study was low, but the risk of bias due to confounding was high (Supplementary Table 4).

The possibility of publication bias of the studies included in the meta-analysis is less likely according to the symmetry observed in funnel plots (Supplementary Figure 1).

### Cardiometabolic Disorders and Monocyte Subsets (Qualitative Analyses)

#### Overweight and Obesity

Two studies examined the association between overweight (25 ≤ BMI <30 kg/m^2^) and the distribution of monocyte subsets. One study reported significantly higher numbers of CM and IM in subjects with overweight compared to healthy controls (33) (Table 2). The other study reported significantly lower percentage and number of CM but no change in in the percentage and number of IM in subjects with overweight compared to healthy controls (34) (Table 2). Both studies reported no change in the number (33, 34) and percentage (34) of NCM in subjects with overweight compared to healthy controls (Table 2).

Six studies examined the association between obesity (BMI ≥ 30 kg/m^2^) and the distribution of monocyte subsets. Studies quantifying the number of CM reported inconsistent results with two studies showing a significantly lower number of CM (34, 37) and one study showing a significantly higher number of CM (33) in subjects with obesity compared to healthy controls (Table 2). However, three studies quantifying the percentage of CM reported a significantly lower percentage of CM in subjects with obesity compared to healthy subjects (34, 36, 38) (Table 2). Five studies reported a significantly higher percentage (34, 36–38) and number (33, 34, 36, 37) of IM in subjects with obesity compared to healthy controls (Table 2). Three studies reported significantly higher percentage (34, 37) and number (34, 36, 37) of NCM in subject with obesity compared to healthy controls (Table 2).

#### Metabolic Syndrome

Two studies investigated the association between metabolic syndrome and the distribution of monocyte subsets. Both studies reported significantly higher number (39) and percentage (40) of NCM and no change in the number and percentage of IM (39, 40) in subjects with metabolic syndrome compared to healthy controls (Table 2). Among them, one study reported the percentage of CM is significantly lower in subjects with metabolic syndrome compared to healthy controls (39) and the other study reported no significant change (40) (Table 2).

#### Hypercholesterolemia

Two studies reported an association between hypercholesterolemia and the distribution of monocyte subsets. One study found the percentage of IM is higher in subjects with hypercholesterolemia compared to healthy controls (42) (Table 2). The other study reported no association between hypercholesterolemia and the percentage of any monocyte subset (41) (Table 2). However, significant phenotypic changes were observed in each monocyte subset, i.e., a higher IL6R, CD49d, and CXCR4 expression on CM; a higher IL6R and CXCR4 expression on IM; a higher CXCR4 expression on NCM (41) (Table 2). Moreover, the number of CM was found to be a significant predictor of carotid stenosis, intima-media thickness and grade 2 neovascularization (41) (Table 2).

#### Type 2 Diabetes

Four studies examined the relationship between type 2 diabetes and the distribution of monocyte subsets. Three studies reported that the number (34) and the percentage (34, 44, 45) of CM are significantly lower, and the number and the percentage of IM (34) and NCM (34, 44) are significantly higher in subjects with type 2 diabetes compared to healthy controls (Table 2). In contrast, one study reported a significantly lower number of NCM in subjects with type 2 diabetes, but also reported a significantly higher mean fluorescence intensity (MFI) of CD11c on NCM in subjects with type 2 diabetes compared to healthy controls (43) (Table 2).

### Cardiovascular Disease and Monocyte Subsets (Qualitative Analysis)

#### Atherosclerosis

Three studies examined the association between atherosclerosis and the distribution of monocyte subsets. All three studies reported that the percentage of IM was significantly higher in patients with atherosclerosis compared to healthy controls (27, 46, 47) (Table 3). One study reported a higher percentage of CM (46), another study reported a lower percentage of CM (47), and the other study reported no change in the percentage of CM (27) in patients with atherosclerosis compared to healthy controls (Table 3). There was no significant change in the percentage of NCM in subjects with atherosclerosis compared to healthy controls (27, 46, 47) (Table 3).

**Table 3:** Included studies examining the association between the distribution of monocyte subsets and cardiovascular diseases.

**Reference**	**Study design^a^**	**Subjects**			**Outcomes**				
		**Clinical conditions**	***N* (Male %)**	**Age^b^**	**Distribution of monocyte subsets^c^**				**Other outcomes related to monocytes^d^**
					**Unit**	**CM**	**IM**	**NCM**	
**Atherosclerosis (AS)**									
Chelombitko et al. (46)	CSS	Healthy AS	15 (ND) 25 (ND)	25 55	%	↑	↑	~	– The expression of CX3CR1 on IM and NCM was higher in patients with AS compared to healthy controls, suggesting a pre-activated state of monocytes in atherosclerosis.
Williams et al. (27)	CSS	Healthy AS	33 (52) 31 (65)	50 68	%	~	↑	~	– Patients with AS had a higher CD86/CD163 ratio, indicating a more inflammatory monocyte phenotype in atherosclerosis. – In CM, CD163 expression was positively associated with ApoA1, and CD86/CD163 ratio was negatively associated with ApoA1 and HDL cholesterol.
Xiang et al. (47)	CSS	Healthy Coronary AS	112 (53) 110 (49)	58 57	%	↓	↑	~	– The number of monocytes was higher in patients with coronary AS compared to healthy controls.
**Coronary artery/heart disease (CAD/CHD)**									
Czepluch et al. (48)	CSS	Healthy CAD	18 (78) 52 (77)	58 70	%	~	~	~	– The expression of KLF4, which involves in lowering vascular inflammation, was reduced on all monocyte subsets in patients with CAD compared to healthy controls. – KLF4 expressing monocytes were negatively correlated with plasma TNF-α.
Czepluch et al. (49)	CSS	Healthy CAD	64 (72) 60 (77)	54 70	% Number	↑ ~	~ ~	↓ ↓	– Patients with CAD had a higher MPA proportion on all three monocyte subsets and a lower expression of CCR5 on all three monocyte subsets compared to healthy controls.
Jaipersad et al. (41)	CSS	Healthy CAD w/ carotid stenosis	40 (43) 40 (60)	67 70	Number	↑	~	~	– Patients with CAD and carotid stenosis had a higher IL6R and CXCR4 expression on CM and IM, a higher VEGF expression on IM and NCM, and a higher Tie2 expression on all three monocyte subsets compared to healthy controls, suggesting a higher surface expression of receptors implicating angiogenesis and tissue remodeling. – The number of CM significantly predicts carotid stenosis, intima-medial thickness, and grade 2 neovascularization.
Jaipersad et al. (41)	CSS	Healthy CAD w/o carotid stenosis	40 (43) 40 (68)	67 69	Number	↑	~	↑	– Patients with CAD had a higher IL6R expression on CM and IM, a higher CD49d and Tie2 expression on all three monocyte subsets compared to healthy controls. – The number of CM significantly predicts carotid stenosis, intima-medial thickness, and grade 2 neovascularization.
Shantsilaet al. (50)	CSS	Healthy CAD	50 (66) 53 (72)	61 64	Number	~	~	~	– Patients with CAD had a higher IL6R expression on CM and IM compared to healthy controls, suggesting CM and IM may be responsible for IL6R-mediated atherogenesis. – Patients with CAD had a higher expression CXCR4 on CM and NCM compared to healthy controls, suggesting CM and NCM may be mobilized to the tissues in a CXCR4-dependent manner. – Patients with CAD had a higher expression of CD34 on all three monocytes compared to healthy controls, suggesting an enhancement of the angiogenic process in response to the low-grade ischemia present in stable CAD.
Tallone et al. (51)	CSS	Healthy CAD	13 (38) 14 (64)	59 60	%	↓	~	↑	– Patients with CAD had a higher CCR2 and CX3CR1 expression on CM, suggesting an elevated adhesion and accumulation of CM to vascular endothelium.
Tapp et al. (52)	CSS	Healthy	40 (80)	60	%	~	~	~	-
		CAD	40 (83)	60	Number	~	~	~	
Zhou et al. (53)	CSS	Healthy	35 (69)	59	Number	~	~	~	-
		CHD	60 (68)	61					
**Chronic/acute heart failure (CHF/AHF)**									
Amir et al. (54)	CSS	Healthy CHF	29 (52) 59 (76)	60 58	%	↓	~	↑	– Patients with CHF had a higher intracellular IL-13 concentration in IM compared to healthy controls and the percentage of IM was positively correlated with serum IL-13, suggesting that IL-13 and IM may play a role in the process in heart failure.
Van Craenenbroeck et al. (55)	CSS	Healthy CHF	15 (60) 20 (65)	44 51	% Number	~ ↑	~ ~	~ ↑	-
Goonewardena et al. (56)	CSS	Healthy AHF at admission	11 (73) 19 (79)	60 56	% Number	↓ ↑	↑ ↑	↑ ↑	– The number of monocytes was higher in patients with AHF compared to healthy controls.
**Acute myocardial infarction (AMI)/Acute coronary syndrome (ACS)**									
Kazimierczyk et al. (57)	CSS	Healthy	18 (78)	57	%	↓	~	~	-
		AMI at admission (before pPCI, median time of 4 h after onset)	18 (78)	65					
Tapp et al. (52)	CSS	Healthy	40 (80)	60	%	~	↑	↑	-
		AMI at admission (after pPCI, first 24 h)	50 (86)	58	Number	↑	↑	~	
Zhou et al. (53)	CSS	Healthy AMI at admission (after pPCI, first 24 h)	35 (69) 100 (78)	59 60	Number	↑	↑	~	– Patients with AMI had a higher MPA proportion in CM and IM compared to healthy controls.
Zhu et al. (58)	CSS	Healthy ACS (unstable angina + AMI)	27 (81) 68 (79)	63 66	% Number	↑ ↑	↓ ~	~ ↑	– Patients with ACS had a higher percentage and number of monocytes compared to healthy controls. – The number of CM was positively correlated with serum concentrations of lactate dehydrogenase, creatine kinase, myoglobin, suggesting that CM may play a proinflammatory role in the acute onset of ACS. – The percentage of IM was negatively correlated with serum myoglobin levels, which is a myocardial injury marker. – The number of NCM was positively correlated with the serum levels of lactate dehydrogenase, creatine kinase, myoglobin.

a *Study design: CSS; cross-sectional study*.

b *Age: is presented as in the original article (mean or median)*.

c *Distribution of monocyte subsets: Unit number, the absolute counts of monocyte subsets (cells/μl); Unit %, the percent distribution of monocyte subsets; ↑/↓ monocyte subsets in subjects with cardiovascular disease are significantly higher/lower compared to healthy control*.

d *Other outcomes related to monocytes: Outcomes with significant difference are presented*.

*ApoA1, apolipoprotein A1; CCR, C-C Motif Chemokine Receptor; CM, classical monocytes; CXCR4, C-X-C chemokine receptor type 4; CX3CR1, CX3C chemokine receptor 1; HDL, high-density lipoprotein; IL, interleukin; IL6R, interleukin 6 receptor; IM, intermediate monocytes; KLF, Krüppel-like factor 4; MPA, monocyte-platelet aggregate; NCM, non-classical monocytes; ND, No data; pPCI, primary percutaneous coronary intervention; Tie, TEK receptor tyrosine kinase; TLR, Toll-like receptor; TNF, Tumor Necrosis Factor; VEGF, Vascular endothelial growth factor*.

#### Coronary Artery/Heart Disease

Six studies examined the association between coronary artery disease and the distribution of monocyte subsets. No change was observed in the number and the percentage of IM in subjects with coronary artery disease compared to healthy controls (41, 48–52) (Table 3). Mixed results were reported for CM and NCM. Two studies reported a higher number (41) and percentage (49) of CM, and one study reported a lower percentage of CM (51) in patients with coronary artery disease compared to healthy controls (Table 3). One study reported a lower number and percentage of NCM (49), whereas two studies reported a higher number (41) and percentage (51) of NCM in patients with coronary artery disease compared to healthy controls (Table 3). However, significant phenotypic changes among the monocyte subsets were reported in subjects with coronary artery disease compared to healthy controls, i.e., a higher proportion of monocyte-platelet aggregate (MPA) on all three monocyte subsets (49); a higher IL6R expression on CM and IM (41, 50); a higher TLR4 and Tie2 expression in NCM (41) (Table 3).

One study examined the association between coronary heart disease and the distribution of monocyte subsets. No change was observed in the number of any monocyte subset in patients with coronary heart disease compared to healthy controls (53) (Table 3).

#### Chronic/Acute Heart Failure

Two studies examined the association between chronic heart failure and the distribution of monocyte subsets. One study reported a lower percentage of CM (54), while the other study reported a higher number of CM (55) in patients with chronic heart failure compared to healthy controls (Table 3). Both studies reported a higher number (55) and percentage (54) of NCM, and no change in IM in patients with chronic heart failure compared to healthy controls (Table 3).

One study investigated the association between acute heart failure and the distribution of monocyte subsets. The numbers and percentages of IM and NCM were significantly higher in patients with acute heart failure at hospital admission compared to healthy controls (56) (Table 3). This study also reported a lower percentage of CM and a higher number of CM in patients with acute heart failure compared to healthy controls (56) (Table 3).

#### Acute Myocardial Infarction/Acute Coronary Syndrome

Three studies investigated the association between acute myocardial infarction and the distribution of monocyte subsets. Two studies demonstrated a significantly higher number of CM (52, 53), higher number (52, 53) and percentage (52) of IM, and a higher percentage of NCM (52) in patients with myocardial infarction compared to healthy controls, when the monocyte subsets were assessed during the first 24-h after primary percutaneous coronary intervention (pPCI) (Table 3). One study examined the distribution of monocyte subsets before pPCI at median time of 4-h after the onset of acute myocardial infarction, and found a lower percentage of CM but no alterations in IM and NCM in patients with acute myocardial infarction compared to healthy subjects (57) (Table 3).

One study examined the association between acute coronary syndrome and the distribution of monocyte subsets. The number and the percentage of CM were higher, the percentage of IM was lower, and the number of NCM was higher in subjects with acute coronary syndrome compared to healthy controls (58) (Table 3).

### Cardiometabolic Disorders and Monocyte Subsets (Meta-Analysis)

**Figure 2:**
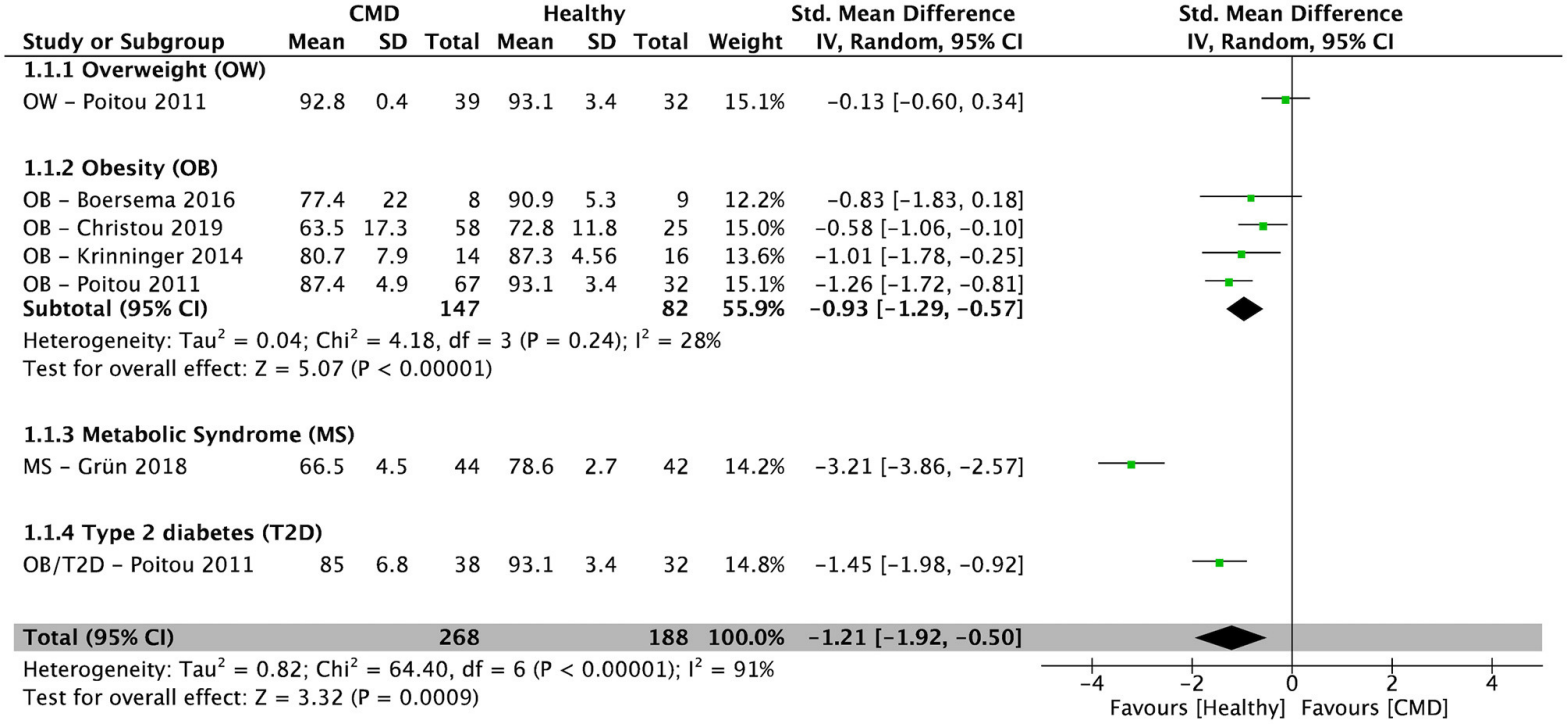
Random-effects meta-analysis of standardized mean difference in the percentage of classical monocytes in subjects with cardiometabolic disorders compared to healthy controls. CI, confidence interval; CMD, cardiometabolic disorders; SD, standard deviation.

After pooling the results from five cross-sectional studies (34–36, 38, 39), there was a significantly lower percentage of CM in individuals with cardiometabolic disorders (overweight, obesity, metabolic syndrome, and type 2 diabetes) when compared to healthy controls [SMD = −1.21; 95% CI (−1.92, −0.50); *P* = 0.0009; *I*^2^ = 91%] (Figure 2). In subjects with obesity, the pooled percentage of CM from four cross-sectional studies (34–36, 38) was significantly lower compared to healthy controls [SMD = −0.93; 95% CI (−1.29, −0.57); *P* < 0.00001; *I*^2^ = 28%] (Figure 2).

**Figure 3:**
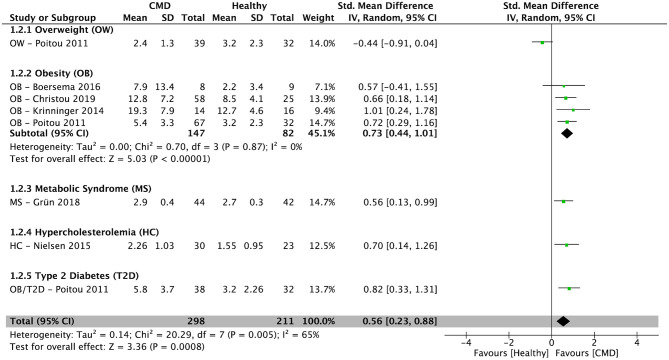
Random-effects meta-analysis of standardized mean difference in the percentage of intermediate monocytes in subjects with cardiometabolic disorders compared to healthy controls. CI, confidence interval; CMD, cardiometabolic disorders; SD, standard deviation.

After pooling results from six cross-sectional studies (34–36, 38, 39, 42), there was a significantly higher percentage of IM in subjects with cardiometabolic disorders compared to healthy controls [SMD = 0.56; 95% CI (0.23, 0.88); *P* = 0.0008; *I*^2^ = 65%] (Figure 3). In subjects with obesity, the pooled percentage of IM from four cross-sectional studies (34–36, 38) was significantly higher compared to healthy controls [SMD = 0.73; 95% CI (0.44, 1.01); *P* < 0.00001; *I*^2^ = 0%] (Figure 3).

**Figure 4:**
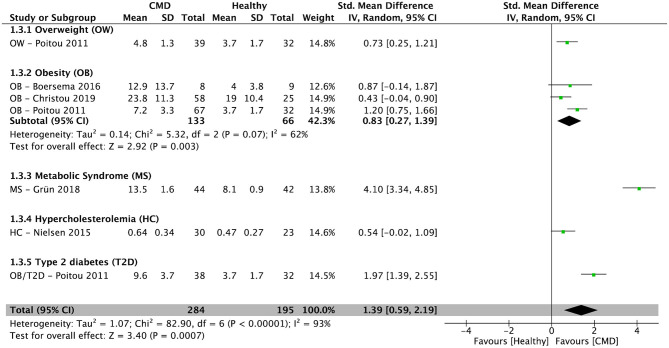
Random-effects meta-analysis of standardized mean difference in the percentage of non-classical monocytes in subjects with cardiometabolic disorders compared to healthy controls. CI, confidence interval; CMD, cardiometabolic disorders; SD, standard deviation.

After pooling results from five cross-sectional studies (34–36, 39, 42), there was a significantly higher percentage of NCM in subjects with cardiometabolic disorders compared to healthy controls [SMD = 1.39; 95% CI (0.59, 2.19); *P* = 0.0007; *I*^2^ = 93%] (Figure 4). In subjects with obesity, the pooled percentage of NCM from three cross-sectional studies (34–36) was significantly higher compared to healthy controls [SMD = 0.83; 95% CI (0.27, 1.39); *P* = 0.003; *I*^2^ = 62%] (Figure 4).

Six cross-sectional studies report the correlation between each monocyte subset and clinical parameters (33, 34, 36, 39, 44, 45). There was a positive correlation between the percentage of CM and fat mass [*r* = 0.358; 95% CI (0.21, 0.49); *P* < 0.001, *I*^2^ = 0%] (Supplementary Table 5). The percentage of IM was positively correlated with age [*r* = 0.204; 95% CI (0.01, 0.39); *P* = 0.043, *I*^2^ = 0%], BMI [*r* = 0.225; 95% CI (0.001, 0.43); *P* = 0.049, *I*^2^ = 81%], fat mass [*r* = 0.311; 95% CI (0.08, 0.51); *P* = 0.009, *I*^2^ = 75%], FBG [*r* = 0.149; 95% CI (0.04, 0.25); *P* = 0.006, *I*^2^ = 0%], fasting insulin [*r* = 0.227; 95% CI (0.11, 0.34); *P* < 0.001, *I*^2^ = 0%], HOMA-IR [*r* = 0.236; 95% CI (0.12, 0.35); *P* < 0.001, *I*^2^ = 0%], TG [*r* = 0.288; 95% CI (0.12, 0.44); *P* = 0.001, *I*^2^ = 52%], and CRP [*r* = 0.285; 95% CI (0.13, 0.43); *P* = 0.001, *I*^2^ = 53%] (Supplementary Table 5). The percentage of NCM was positively correlated with BMI [*r* = 0.257; 95% CI (0.02, 0.47); *P* = 0.04, *I*^2^ = 77%], fasting insulin [*r* = 0.338; 95% CI (0.09, 0.55); *P* = 0.008, *I*^2^ = 72%], and HOMA-IR [*r* = 0.325; 95% CI (0.06, 0.55); *P* = 0.018, *I*^2^ = 76%], and negatively correlated with HDL-C [*r* = −0.260; 95% CI (−0.45, −0.06); *P* = 0.013, *I*^2^ = 69%] (Supplementary Table 5).

## Discussion

To our knowledge, this is the first systematic review and meta-analysis that has evaluated the evidence linking either cardiometabolic disorders or CVD with the distribution of monocyte subsets. We found that 9 out of 13 studies demonstrated a higher percentage and number of IM and/or NCM in subjects with cardiometabolic disorders (overweight/obesity, metabolic syndrome, hypercholesterolemia, and type 2 diabetes) compared to healthy controls. Moreover, we found that 11 out of 15 studies reported a higher percentage and number of IM and/or NCM in subjects with CVD (atherosclerosis, coronary artery/heart disease, chronic/acute heart failure, acute myocardial infarction and acute coronary syndrome) compared to healthy controls. In the meta-analysis, we demonstrated that subjects with cardiometabolic disorders had a significantly higher percentage of IM and NCM and a significantly lower percentage of CM compared to healthy controls. Collectively, results from both the systematic review and meta-analysis suggest that the distribution of monocytes may be altered resulting in a higher percentage and number of IM and NCM in subjects with cardiometabolic disorders and CVD. However, additional studies with larger sample sizes and more diverse populations are needed to better understand this relationship.

Monocytes play a crucial role in the pathology of atherosclerosis and CVD (59). In epidemiological and clinical studies, monocytosis is an independent risk factor for CVD (60–62). However, our understanding of the role of monocyte subsets in the development of CVD is largely based on animal models of atherosclerosis and myocardial infarction (63, 64). Mouse CM (Gr1^+^/Ly-6C^high^; CD14^++^CD16^−^ homolog in humans) are recruited into the intima, differentiate into macrophages that phagocytose lipids and cholesterol derivatives, forming foam cells in the subintima, which ultimately contribute to the accumulation of fatty deposits (plaques) (63, 64). Mouse NCM (Gr1^−^/Ly-6C^low^; CD14^+^CD16^++^ homolog in humans) are involved in scavenging the endothelium and clearing lipid derivatives, and dead and dying cells (63, 64). In humans, three distinct monocytes were defined in 2010 and includes a third subset called IM in addition to CM and NCM (16). Each monocyte subset appears to have distinct phenotypic and functional properties that may contribute differentially to inflammatory responses (16).

CM comprise the largest proportion of circulating monocytes (80–90%) (65). Based on the proatherogenic function of CM observed in mouse models, they have been hypothesized to be the subset involved in the pathogenesis of CVD (64). In humans, CM highly express cell surface receptors that are involved in pathogen recognition, phagocytosis, and adhesion and migration into the vascular endothelium (17). In our systematic review, six out of eight studies quantifying the number of monocyte subsets reported a higher number of CM in subjects with CVD. However, despite the increase in total number of CM, five out of 12 studies quantifying the percentage of monocyte subsets reported a lower percentage of CM in subjects with CVD. In addition, our meta-analysis demonstrated a significantly lower percentage of CM in subjects with obesity, metabolic syndrome, and type 2 diabetes compared to healthy controls. Combined these results suggests that despite a potential overall increase in the number of CM, a shift in the distribution of monocyte subsets from CM to IM and/or NCM may occur in subjects with both cardiometabolic disorders and CVD. This shift may influence atherosclerotic risk or progression, and should be explored in future studies.

In humans, evidence to date suggests that IM and NCM account for 10–20% of circulating monocytes (65) and they may be linked to atherosclerosis and CVD. *In vivo* LPS challenge studies in humans demonstrate an expansion of IM and NCM 24 h after LPS injection (2 ng/kg body weight) in healthy subjects (66, 67), suggesting that IM and NCM IM and NCM respond acutely to a standardized low-grade inflammatory stimulus. IM produce and secrete high levels of proinflammatory cytokines IL-1β, IL-6, and TNF-α upon LPS stimulation (18, 19, 68). IM also play role in angiogenesis, antigen presentation, and proliferation and stimulation of T cells (17, 18). Our systematic review found that five out of six studies reported a higher percentage and number of IM in subjects with overweight/obesity (33, 34, 36–38), one out of two studies reported a higher percentage of IM in subjects with hypercholesterolemia (42), but only one out of four studies reported higher percentage and number of IM in subjects with type 2 diabetes (34) compared to healthy controls. Our meta-analysis demonstrated a significantly higher percentage of IM in subjects with obesity, metabolic syndrome, hypercholesterolemia and type 2 diabetes compared to healthy controls. In addition, in the meta-analysis of correlation coefficients, we demonstrated a positive correlation between IM and CVD risk factors including BMI, fat mass, FBG, fasting insulin, HOMA-IR, TG and CRP. Moreover, previous studies report that weight loss after gastric bypass surgery in patients with obesity leads to a significant reduction in IM (34, 69). Combined these finding suggest that an increase in IM is observed in subjects with cardiometabolic disorders, particularly those with obesity, and dyslipidemia and possibly those with hyperglycemia.

In addition to the relationship between IM and the previously discussed cardiometabolic disorders, emerging evidence suggest that an increase in IM may be observed in subjects with CVD. Two prospective cohort studies identified the number of IM as a positive predictor of major cardiovascular events in patients referred for elective coronary angiography (*n* = 951) (26) and in patients with chronic kidney disease (*n* = 438) (70). In our systematic review, we found that two studies reported a higher percentage of IM in subjects with atherosclerosis (27, 46), one study reported higher percentage and number of IM in subjects with acute heart failure (56), and two studies reported higher percentage and number of IM in patients with acute myocardial infarction (52, 53) compared to healthy controls. Although no correlation between IM and coronary artery/heart disease was reported, there were phenotypic changes in IM in these patients such as a higher percentage of MPA (49) [a sensitive indicator of monocyte inflammation (71)] and a higher expression of Tie2 (41) [an indicator of elevated proatherogenic activity of this monocyte subsets (72, 73)] compared to healthy controls. Data from our systematic review and meta-analysis in combination with findings from other studies suggest that an elevated percentage of IM is observed in subjects with cardiometabolic disorders and CVD. Additional studies are warranted to determine if the percentage of IM are altered as a consequence of cardiometabolic dysfunction and/or CVD, and/or if the elevated percentage of IM contributes to the risk or progression of cardiometabolic disorders and/or CVD.

NCM patrol along the vascular endothelium and selectively detect damaged cells (74). However, conflicting results exists with some reports demonstrating an atheroprotective role while others report a proatherogenic role of NCM. In support of the atheroprotective role of NCM, when Nr4a1^−/−^ mice that lack Ly6C^lo^ NCM are crossed with the atherogenic ApoE^−/−^ mice and fed a Western diet, they have a significant increase in atherosclerotic lesion size compared to ApoE^−/−^ mice with functional NCM (75). Furthermore, when the bone marrow from Nr4a1^−/−^-deficient mice is transplanted into Ldlr^−/−^ mice fed a high-fat diet, these mice develop a larger atherosclerotic lesion size compared to Ldlr^−/−^ mice transplanted with wild-type bone marrow (76). In addition, an expansion of Slan^+^ NCM, which are involved in efferocytosis, is reported in patients with coronary artery disease (77). This expand population of Slan^+^ NCM in subjects with coronary artery disease may reflect a compensatory increase in NCM in response to vascular inflammation, which may suggest an atheroprotective role of NCM. In contrast, Ong et al. report that NCM produce higher basal levels of IL-1β, IL-6, and TNF-α compared to CM (3), suggesting that NCM may contribute to chronic low-grade inflammation. Moreover, TLR8-induced secretion of IL-1β and CCL5 from NCM is higher in subjects with obesity compared to healthy controls (37), suggesting that obesity may enhance the inflammatory phenotype of NCM. Our meta-analysis demonstrated a higher percentage of NCM in subjects with overweight/obesity, metabolic syndrome, hypercholesterolemia and type 2 diabetes compared to healthy subjects. We also demonstrated a positive correlation between NCM and the CVD risk factors including BMI, fasting insulin and HOMA-IR. In our systematic review, we found studies that reported an elevated percentage and number of NCM in subjects with obesity (three out of five studies) (34, 36, 37), metabolic syndrome (two out of two studies) (39, 40), and type 2 diabetes (two out of four studies) (34, 44) compared to healthy controls. Moreover, we found studies that reported a higher percentage of NCM in subjects with coronary artery/heart disease (two out of seven studies), chronic/acute heart failure (three out of three studies), and acute myocardial infarction (one out of three studies) compared to healthy controls. Thus, our results suggest that an elevation in NCM may be observed in subjects with cardiometabolic disorders and some forms of CVD. It is plausible that the presence of NCM in healthy subjects may have an atheroprotective role by patrolling vascular endothelium and detecting damaged cells, but their inflammatory phenotype may be enhanced in those individuals with cardiometabolic disorders and CVD. Future studies are needed to explore the relationship between these observations.

Previous studies demonstrate that inherited (e.g., ethnicity, gender, and age) and environmental (e.g., diet and exercise) factors can influence the distribution of monocyte subsets [reviewed in (78)]. For example, Caucasian populations are reported to have a higher percentage of CM and a lower percentage of NCM compared to African populations (79). Additionally, women are reported to have a lower percentage of circulating NCM compared to men (80). Older adults have a decreased percentage of CM and an increased percentage of IM and NCM (80). Consumption of a single high-fat meal (40) and overall diet quality (81) alters the distribution of NCM and IM, respectively. Lastly, acute bout of exercise (82–85), as well as chronic exercise training (86) alters the distribution of monocyte subsets. Thus, inherited and environmental factors may be confounding factors that influence the distribution of monocyte subsets.

A major strength of our review is that this is the first systematic review and meta-analysis to assess the change in the distribution of monocyte subsets in individuals with cardiometabolic disorders and CVD compared to healthy individuals. This systematic review implemented a search strategy which was comprehensive and complete. Examination of funnel plots revealed minimal evidence of publication bias. Moreover, the meta-analysis had low to moderate heterogeneity observed among studies. Studies in this review include subjects with a mean/median age (20–70 years) and include both male and female subjects, representing a fairly heterogeneous population. However, a limitation of this systematic review is that many studies included in this systematic review did not adjust the outcomes (the percentage and number of monocyte subsets) for potential confounding factors [with the exception of two studies that adjusted monocyte outcomes for age and gender (34, 54)]. Additionally, many of the studies included in the review had a small sample size, and a small number of studies are included in both the systematic review and meta-analysis. Thus, these results should be interpreted with caution. Lastly, variation may exist in defining monocyte subsets using flow cytometric analyses which could significantly impact interpretation of our findings (78).

In conclusion, we demonstrated that a shift in the distribution of monocyte subsets from CM toward IM and NCM may occur in individuals with cardiometabolic disorders and CVD. However, there is insufficient evidence to draw conclusions as to the underlying causality of this association. Future longitudinal studies are needed to demonstrate a causal relationship between the shift in the distribution of monocyte subsets and the risk and/or progression of cardiometabolic disorders and/or CVD.

## Data Availability Statement

The original contributions generated in the study are included in the article/Supplementary Material, further inquiries can be directed to the corresponding author/s.

## Author Contributions

EO and CR designed the research, conducted the research, and collected data. EO, MN, and CR participated in data analysis and interpretation and wrote the paper. CR had primary responsibility for the final content. All authors contributed to the article and approved the submitted version.

## Conflict of Interest

The authors declare that the research was conducted in the absence of any commercial or financial relationships that could be construed as a potential conflict of interest.
