# Supplementary material for: The Association Between Monocyte Subsets and Cardiometabolic Disorders/Cardiovascular Disease: A Systematic Review and Meta-Analysis
Source: Front Cardiovasc Med. 2021 Feb 17;8:640124. doi: 10.3389/fcvm.2021.640124 (PMC7925827; doi:10.3389/fcvm.2021.640124)
Supplement: Supplementary file 1 [file Data_Sheet_1.docx]

Supplementary Material

# Supplementary Tables

## Supplementary Table 1. Preferred Reporting Items for Systematic Reviews and Meta-Analyses (PRISMA) Checklist

| **Section/topic** | **#** | **Checklist item** | **Reported on page #** |
| --- | --- | --- | --- |
| **TITLE** | | |  |
| Title | 1 | Identify the report as a systematic review, meta-analysis, or both. | 1 |
| **ABSTRACT** | | |  |
| Structured summary | 2 | Provide a structured summary including, as applicable: background; objectives; data sources; study eligibility criteria, participants, and interventions; study appraisal and synthesis methods; results; limitations; conclusions and implications of key findings; systematic review registration number. | 1 |
| **INTRODUCTION** | | |  |
| Rationale | 3 | Describe the rationale for the review in the context of what is already known. | 2 |
| Objectives | 4 | Provide an explicit statement of questions being addressed with reference to participants, interventions, comparisons, outcomes, and study design (PICOS). | 2 |
| **METHODS** | | |  |
| Protocol and registration | 5 | Indicate if a review protocol exists, if and where it can be accessed (e.g., Web address), and, if available, provide registration information including registration number. | 2 |
| Eligibility criteria | 6 | Specify study characteristics (e.g., PICOS, length of follow-up) and report characteristics (e.g., years considered, language, publication status) used as criteria for eligibility, giving rationale. | 2 |
| Information sources | 7 | Describe all information sources (e.g., databases with dates of coverage, contact with study authors to identify additional studies) in the search and date last searched. | 2 |
| Search | 8 | Present full electronic search strategy for at least one database, including any limits used, such that it could be repeated. | 2 Supplementary Table 2 |
| Study selection | 9 | State the process for selecting studies (i.e., screening, eligibility, included in systematic review, and, if applicable, included in the meta-analysis). | 2-3 |
| Data collection process | 10 | Describe method of data extraction from reports (e.g., piloted forms, independently, in duplicate) and any processes for obtaining and confirming data from investigators. | 2-3 |
| Data items | 11 | List and define all variables for which data were sought (e.g., PICOS, funding sources) and any assumptions and simplifications made. | 3 |
| Risk of bias in individual studies | 12 | Describe methods used for assessing risk of bias of individual studies (including specification of whether this was done at the study or outcome level), and how this information is to be used in any data synthesis. | 3 |
| Summary measures | 13 | State the principal summary measures (e.g., risk ratio, difference in means). | 3 |
| Synthesis of results | 14 | Describe the methods of handling data and combining results of studies, if done, including measures of consistency (e.g., I^2^) for each meta-analysis. | 3 |
| Risk of bias across studies | 15 | Specify any assessment of risk of bias that may affect the cumulative evidence (e.g., publication bias, selective reporting within studies). | 3 |
| Additional analyses | 16 | Describe methods of additional analyses (e.g., sensitivity or subgroup analyses, meta-regression), if done, indicating which were pre-specified. | 3 |
| **RESULTS** | | | |
| Study selection | 17 | Give numbers of studies screened, assessed for eligibility, and included in the review, with reasons for exclusions at each stage, ideally with a flow diagram. | 3-4 |
| Study characteristics | 18 | For each study, present characteristics for which data were extracted (e.g., study size, PICOS, follow-up period) and provide the citations. | 4 |
| Risk of bias within studies | 19 | Present data on risk of bias of each study and, if available, any outcome level assessment (see item 12). | 4 |
| Results of individual studies | 20 | For all outcomes considered (benefits or harms), present, for each study: (a) simple summary data for each intervention group (b) effect estimates and confidence intervals, ideally with a forest plot. | 5-10 |
| Synthesis of results | 21 | Present results of each meta-analysis done, including confidence intervals and measures of consistency. | 10-11 |
| Risk of bias across studies | 22 | Present results of any assessment of risk of bias across studies (see Item 15). | 4 |
| Additional analysis | 23 | Give results of additional analyses, if done (e.g., sensitivity or subgroup analyses, meta-regression [see Item 16]). | 10 |
| **DISCUSSION** | | | |
| Summary of evidence | 24 | Summarize the main findings including the strength of evidence for each main outcome; consider their relevance to key groups (e.g., healthcare providers, users, and policy makers). | 10 |
| Limitations | 25 | Discuss limitations at study and outcome level (e.g., risk of bias), and at review-level (e.g., incomplete retrieval of identified research, reporting bias). | 13 |
| Conclusions | 26 | Provide a general interpretation of the results in the context of other evidence, and implications for future research. | 13 |
| **FUNDING** | | | |
| Funding | 27 | Describe sources of funding for the systematic review and other support (e.g., supply of data); role of funders for the systematic review. | 13 |

## Supplementary Table 2. Database, search terms, filters and number of results

| **Database**  **(Search Date)** | **Search Terms** | **Filters** | **Number of results** |
| --- | --- | --- | --- |
| CINAHL  (10/9/2020) | (MH "Monocytes") OR TX ("monocyte" OR "monocytes")  AND  TX (“classical” OR “intermediate” OR “non-classical” OR “nonclassical” OR “subset” OR “subsets” OR “subpopulation” OR “subpopulations” OR “sub-population” OR “sub-populations”)  AND  ((MH "Chronic Disease") OR (MH “Noncommunicable Diseases”) OR (MH "Obesity") OR (MH "Metabolic Diseases") OR (MH “Metabolic Syndrome X”) OR (MH "Diabetes Mellitus, Type 2") OR (MH “Hyperglycemia”) OR (MH “Glucose Intolerance”) OR (MH “Insulin Resistance”) OR (MH “Hyperlipidemia”) OR (MH “Hypercholesterolemia”) OR (MH “Cardiovascular Diseases”) OR (MH “Atherosclerosis”) OR (MH “hypertension)  OR TX (“chronic disease” OR “chronic diseases” OR “noncommunicable disease” OR “noncommunicable diseases” OR “non-communicable disease” OR “non-communicable diseases” OR “overweight” OR “obesity” OR “obese” OR “obesogenic” OR “metabolic disease” OR “metabolic diseases” OR “metabolic disorder” OR “metabolic disorders” OR “metabolic abnormality” OR “metabolic abnormalities” OR “metabolic syndrome” OR “metabolic syndromes” OR “metabolic syndrome X” OR “type 2 diabetes” OR “diabetes mellitus type 2” OR “diabetes” OR “diabetic” OR “dysglycemia” OR “dysglycaemia” OR “dysglycemic” OR “dysglycaemic” OR “hyperglycaemia” OR “hyperglycemic” OR “hyperglycaemic” OR “glucose intolerance” OR “glucose intolerant” OR “insulin resistance” OR “insulin resistant” OR “dyslipidemia” OR “dyslipidemias” OR “dyslipidemic” OR “dyslipidaemia” OR “dyslipidaemic” OR “hyperlipidemia” OR “hyperlipidaemia” OR “hyperlipidemic” OR “hyperlipidaemic” OR “hypercholesterolemia” OR “hypercholesterolaemia” OR “hypercholesterolemic” OR “hypercholesterolaemia” OR “hypercholesterolaemic” OR “cardiovascular disease” OR “cardiovascular diseases” OR “atherosclerosis” OR “atherosclerotic” OR “atherogenesis” OR “atherogenic” OR “hypertension” OR “hypertensive” OR “cardiovascular risk” OR “cardiovascular risks” OR “metabolic risk” OR “metabolic risks” OR “cardiometabolic risk” OR “cardiometabolic risks”)) | Language: English | 185 |
| Cochrane Library CENTRAL (10/9/2020) | ("monocytes"[mesh] OR "monocyte"[tw] OR "monocytes"[tw])  AND  (“classical”[tw] OR “intermediate”[tw] OR “non-classical”[tw] OR “nonclassical”[tw] OR “subset”[tw] OR “subsets”[tw] OR “subpopulation”[tw] OR “subpopulations”[tw] OR “sub-population”[tw] OR “sub-populations”[tw])  AND  (“chronic disease”[mesh] OR “chronic disease”[tw] OR “chronic diseases”[tw] OR “noncommunicable disease”[tw] OR “noncommunicable diseases”[tw] OR “non-communicable disease”[tw] OR “non-communicable diseases”[tw] OR “overweight”[mesh] OR “overweight”[tw] OR “obesity”[mesh] OR “obesity”[tw] OR “obese”[tw] OR “obesogenic”[tw] OR “metabolic disease”[tw] OR “metabolic diseases”[tw] OR “metabolic disorder”[tw] OR “metabolic disorders”[tw] OR “metabolic abnormality”[tw] OR “metabolic abnormalities”[tw] OR “metabolic syndrome”[mesh] OR “metabolic syndrome”[tw] OR “metabolic syndromes”[tw] OR “metabolic syndrome X”[tw] OR “Diabetes Mellitus, Type 2”[mesh] OR “diabetes mellitus type 2”[tw] OR “type 2 diabetes”[tw] OR “diabetes”[tw] OR “diabetic”[tw] OR “dysglycemia”[tw] OR “dysglycaemia”[tw] OR “dysglycemic”[tw] OR “dysglycaemic”[tw] OR “hyperglycemia”[mesh] OR “hyperglycaemia”[tw] OR “hyperglycemic”[tw] OR “hyperglycaemic”[tw] OR “glucose intolerance”[mesh] OR “glucose intolerance”[tw] OR “glucose intolerant”[tw] OR “insulin resistance”[mesh] OR “insulin resistance”[tw] OR “insulin resistant”[tw] OR “dyslipidemias”[mesh] OR “dyslipidemia”[tw] OR “dyslipidemias”[tw] OR “dyslipidemic”[tw] OR “dyslipidaemia”[tw] OR “dyslipidaemic”[tw] OR “hyperlipidemia”[tw] OR “hyperlipidaemia”[tw] OR “hyperlipidemic”[tw] OR “hyperlipidaemic”[tw] OR “hypercholesterolemia”[mesh] OR “hypercholesterolemia”[tw] OR “hypercholesterolaemia”[tw] OR “hypercholesterolemic”[tw] OR “hypercholesterolaemia”[tw] OR “hypercholesterolaemic”[tw] OR “cardiovascular diseases”[mesh] OR “cardiovascular disease”[tw] OR “cardiovascular diseases”[tw] OR “atherosclerosis”[mesh] OR “atherosclerosis”[tw] OR “hypertension”[mesh] OR “hypertension”[tw] OR “hypertensive”[tw] OR “cardiovascular risk”[tw] OR “metabolic risk”[tw] OR “metabolic risks”[tw] OR “cardiometabolic risk”[tw] OR “cardiometabolic risks”[tw] OR “cardiovascular risks”[tw]) | - | 159 |
| PubMed  (10/9/2020) | ("monocytes"[mesh] OR "monocyte"[tw] OR "monocytes"[tw])  AND  (“classical”[tw] OR “intermediate”[tw] OR “non-classical”[tw] OR “nonclassical”[tw] OR “subset”[tw] OR “subsets”[tw] OR “subpopulation”[tw] OR “subpopulations”[tw] OR “sub-population”[tw] OR “sub-populations”[tw])  AND  (“chronic disease”[mesh] OR “chronic disease”[tw] OR “chronic diseases”[tw] OR “noncommunicable disease”[tw] OR “noncommunicable diseases”[tw] OR “non-communicable disease”[tw] OR “non-communicable diseases”[tw] OR “overweight”[mesh] OR “overweight”[tw] OR “obesity”[mesh] OR “obesity”[tw] OR “obese”[tw] OR “obesogenic”[tw] OR “metabolic disease”[tw] OR “metabolic diseases”[tw] OR “metabolic disorder”[tw] OR “metabolic disorders”[tw] OR “metabolic abnormality”[tw] OR “metabolic abnormalities”[tw] OR “metabolic syndrome”[mesh] OR “metabolic syndrome”[tw] OR “metabolic syndromes”[tw] OR “metabolic syndrome X”[tw] OR “Diabetes Mellitus, Type 2”[mesh] OR “diabetes mellitus type 2”[tw] OR “type 2 diabetes”[tw] OR “diabetes”[tw] OR “diabetic”[tw] OR “dysglycemia”[tw] OR “dysglycaemia”[tw] OR “dysglycemic”[tw] OR “dysglycaemic”[tw] OR “hyperglycemia”[mesh] OR “hyperglycaemia”[tw] OR “hyperglycemic”[tw] OR “hyperglycaemic”[tw] OR “glucose intolerance”[mesh] OR “glucose intolerance”[tw] OR “glucose intolerant”[tw] OR “insulin resistance”[mesh] OR “insulin resistance”[tw] OR “insulin resistant”[tw] OR “dyslipidemias”[mesh] OR “dyslipidemia”[tw] OR “dyslipidemias”[tw] OR “dyslipidemic”[tw] OR “dyslipidaemia”[tw] OR “dyslipidaemic”[tw] OR “hyperlipidemia”[tw] OR “hyperlipidaemia”[tw] OR “hyperlipidemic”[tw] OR “hyperlipidaemic”[tw] OR “hypercholesterolemia”[mesh] OR “hypercholesterolemia”[tw] OR “hypercholesterolaemia”[tw] OR “hypercholesterolemic”[tw] OR “hypercholesterolaemia”[tw] OR “hypercholesterolaemic”[tw] OR “cardiovascular diseases”[mesh] OR “cardiovascular disease”[tw] OR “cardiovascular diseases”[tw] OR “atherosclerosis”[mesh] OR “atherosclerosis”[tw] OR “hypertension”[mesh] OR “hypertension”[tw] OR “hypertensive”[tw] OR “cardiovascular risk”[tw] OR “metabolic risk”[tw] OR “metabolic risks”[tw] OR “cardiometabolic risk”[tw] OR “cardiometabolic risks”[tw] OR “cardiovascular risks”[tw])  NOT  (“animals”[mesh] NOT (“humans”[mesh] AND “animals”[mesh])) | Language: English | 1542 |

## Supplementary Table 3. Quality of observational studies included in the systematic review

| **Reference** | **Q1** | **Q2** | **Q3** | **Q4** | **Q5** | **Q6** | **Q7** | **Q8** | **Q9** | **Q10** | **Q11** | **Q12** | **Q13** | **Q14** | **Quality** |
| --- | --- | --- | --- | --- | --- | --- | --- | --- | --- | --- | --- | --- | --- | --- | --- |
| Amir, 2012 | Y | Y | NA | Y | N | Y | Y | NA | Y | NA | Y | NR | NA | Y | Good |
| Boersema, 2016^1^ | Y | Y | NA | Y | N | Y | Y | NA | Y | NA | Y | NR | NA | N | Fair |
| Chelombitko, 2014 | Y | N | NA | N | N | Y | Y | NA | Y | NA | Y | NR | NA | N | Fair |
| Christou, 2019^1,2^ | Y | Y | NA | Y | Y | Y | Y | NA | Y | NA | Y | NR | NA | N | Good |
| Czepluch, 2013 | Y | N | NA | N | N | Y | Y | NA | Y | NA | Y | NR | NA | N | Fair |
| Czepluch, 2014 | Y | Y | NA | Y | N | Y | Y | NA | Y | NA | Y | NR | NA | N | Fair |
| Devêvre, 2019 | Y | Y | NA | N | N | Y | Y | NA | Y | NA | Y | NR | NA | N | Fair |
| Friedrich, 2019^2^ | Y | Y | NA | N | N | Y | Y | NA | Y | NA | Y | NR | NA | N | Fair |
| Goonewardena, 2016 | Y | Y | NA | Y | N | Y | Y | NA | Y | NA | Y | NR | NA | N | Fair |
| Grün, 2018^1,2^ | Y | Y | NA | Y | N | Y | Y | NA | Y | NA | Y | NR | NA | N | Fair |
| Jaipersad, 2014 | Y | Y | NA | Y | Y | Y | Y | NA | Y | NA | Y | NR | NA | Y | Good |
| Kazimierczyk, 2019 | Y | Y | NA | Y | N | Y | Y | NA | Y | NA | Y | NR | NA | N | Fair |
| Krinninger, 2014^1^ | Y | Y | NA | Y | N | Y | Y | NA | Y | NA | Y | NR | NA | N | Fair |
| Nielsen, 2015^1^ | Y | Y | NA | Y | N | Y | Y | NA | Y | NA | Y | NR | NA | N | Fair |
| Poitou, 2011^1,2^ | Y | Y | NA | Y | N | Y | Y | NA | Y | NA | Y | NR | NA | Y | Good |
| Shantsila, 2014 | Y | Y | NA | Y | Y | Y | Y | NA | Y | NA | Y | NR | NA | Y | Good |
| Tallone, 2011 | N | Y | NA | Y | N | Y | Y | NA | Y | NA | Y | NR | NA | N | Fair |
| Tapp, 2011 | Y | Y | NA | Y | Y | Y | Y | NA | Y | NA | Y | NR | Y | Y | Good |
| Valtierra-Alvarado, 2020^2^ | Y | Y | NA | Y | N | Y | Y | NA | Y | NA | Y | NR | NA | N | Fair |
| Van Craenenbroeck, 2014 | Y | Y | NA | Y | N | Y | Y | NA | Y | NA | Y | N | NA | Y | Good |
| von Scholten, 2016 | Y | Y | NA | Y | N | Y | Y | NA | Y | NA | Y | NR | NA | Y | Good |
| Williams, 2017 | Y | Y | NA | Y | N | Y | Y | NA | Y | NA | Y | NR | NA | N | Fair |
| Xiang, 2020 | Y | N | NA | Y | N | Y | Y | NA | Y | NA | Y | NR | NA | N | Poor |
| Zaharieva, 2017^2^ | Y | Y | NA | N | N | Y | Y | NA | Y | NA | Y | NR | NA | N | Fair |
| Zhou, 2016 | Y | Y | NA | Y | N | Y | Y | NA | Y | NA | Y | NR | NA | N | Fair |
| Zhu, 2015 | N | N | NA | N | N | Y | Y | NA | Y | NA | Y | NR | NA | N | Fair |

^1^Studies included in meta-analysis evaluating the association between monocyte subsets and cardiometabolic disorders. ^2^Studies included in meta-analysis evaluating the correlation coefficients between monocyte subsets and clinical parameters. Abbreviations: N, no; NA, not applicable; NR, not reported; Q, Question; Y, yes. Quality of included studies was assessed using the National Institutes of Health (NIH) Quality Assessment tool for Observational Cohort and Cross-Sectional Studies (https://www.nhlbi.nih.gov/health-pro/guidelines/in-develop/cardiovascular-risk-reduction/tools/cohort). Q1. Was the research question or objective in this paper clearly stated? Q2. Was the study population clearly specified and defined? Q3. Was the participation rate of eligible persons at least 50%? Q4. Were all the subjects selected or recruited from the same or similar populations (including the same time period)? Were inclusion and exclusion criteria for being in the study prespecified and applied uniformly to all participants? Q5. Was a sample size justification, power description, or variance and effect estimates provided? Q6. For the analyses in this paper, were the exposure(s) of interest measured prior to the outcome(s) being measured? Q7. Was the timeframe sufficient so that one could reasonably expect to see an association between exposure and outcome if it existed? Q8. For exposures that can vary in amount or level, did the study examine different levels of the exposure as related to the outcome (e.g., categories of exposure, or exposure measured as continuous variable)? Q9. Were the exposure measures (independent variables) clearly defined, valid, reliable, and implemented consistently across all study participants? Q10. Was the exposure(s) assessed more than once over time? Q11. Were the outcome measures (dependent variables) clearly defined, valid, reliable, and implemented consistently across all study participants? Q12. Were the outcome assessors blinded to the exposure status of participants? Q13. Was loss to follow-up after baseline 20% or less? Q14. Were key potential confounding variables measured and adjusted statistically for their impact on the relationship between exposure(s) and outcome(s)?

## Supplementary Table 4. Quality of non-randomized studies included in the systematic review

| **Reference** | **Bias due to confounding** | **Bias in selection of participants into the study** | **Bias in classification of interventions** | **Bias due to deviations from intended interventions** | **Bias due to missing data** | **Bias in measurement of outcomes** | **Bias in selection of the reported result** | **Overall bias** |
| --- | --- | --- | --- | --- | --- | --- | --- | --- |
| Khan, 2016 | High | Low | Low | Low | Low | Low | Low | Low |

## Supplementary Table 5. Meta-analysis of correlation coefficients between monocyte subsets and clinical parameters

| **Variables** | **N studies** | **Correlation coefficient** | **95% CI** | ***P*-value** | **Heterogeneity** | | | **Reference** |
| --- | --- | --- | --- | --- | --- | --- | --- | --- |
|  |  |  |  |  | **Model** | ***P*-value** | $\mathbf{I}^{\mathbf{2}}$ |  |
| **Classical monocytes** | | | | | | | | |
| Age | 2 | 0.160 | –0.04, 0.35 | 0.114 | R | <0.001 | 88 | Christou 2019, Grün 2018 |
| BMI | 5 | 0.030 | –0.30, 0.36 | 0.865 | R | 0.002 | 80 | Christou 2019, Friedrich 2019, Grün 2018, Zaharieva 2017, Valtierra-Alvarado 2020 |
| Waist circumference | 4 | 0.157 | –0.15, 0.43 | 0.308 | R | 0.002 | 79 | Christou 2019, Friedrich 2019, Grün 2018, Zaharieva 2017 |
| Fat mass (%) | 2 | 0.358 | 0.21, 0.49 | <0.001 | R | 0.327 | 0 | Friedrich 2019, Grün 2018 |
| FBG | 4 | -0.048 | –0.31, 0.22 | 0.728 | R | 0.025 | 68 | Christou 2019, Grün 2018, Zaharieva 2017, Valtierra-Alvarado 2020 |
| HbA1c | 4 | -0.062 | –0.41, 0.30 | 0.744 | R | <0.001 | 87 | Christou 2019, Friedrich 2019, Zaharieva 2017, Valtierra-Alvarado 2020 |
| Fasting insulin | 2 | 0.114 | –0.22, 0.42 | 0.503 | R | 0.095 | 64 | Christou 2019, Grün 2018 |
| HOMA-IR | 2 | 0.136 | –0.07, 0.33 | 0.189 | R | 0.307 | 4 | Christou 2019, Grün 2018 |
| TG | 5 | 0.006 | –0.18, 0.19 | 0.945 | R | 0.057 | 56 | Christou 2019, Friedrich 2019, Grün 2018, Zaharieva 2017, Valtierra-Alvarado 2020 |
| TC | 4 | –0.031 | –0.19, 0.13 | 0.711 | R | 0.232 | 30 | Christou 2019, Friedrich 2019, Grün 2018, Zaharieva 2017 |
| LDL-C | 3 | –0.053 | –0.32, 0.23 | 0.714 | R | 0.092 | 58 | Christou 2019, Grün 2018, Zaharieva 2017 |
| HDL-C | 5 | 0.129 | –0.17, 0.40 | 0.389 | R | <0.001 | 83 | Christou 2019, Friedrich 2019, Grün 2018, Zaharieva 2017, Valtierra-Alvarado 2020 |
| VLDL | 2 | –0.173 | –0.38, 0.05 | 0.125 | R | 0.742 | 0 | Zaharieva 2017, Valtierra-Alvarado 2020 |
| CRP | 3 | 0.127 | –0.26, 0.48 | 0.525 | R | 0.002 | 84 | Christou 2019, Friedrich 2019, Zaharieva 2017 |
| **Intermediate monocytes** | | | | | | | | |
| Age | 2 | 0.204 | 0.01, 0.39 | 0.043 | R | 0.996 | 0 | Christou 2019, Grün 2018 |
| BMI | 6 | 0.225 | 0.001, 0.43 | 0.049 | R | <0.001 | 81 | Christou 2019, Friedrich 2019, Grün 2018, Poitou 2011, Zaharieva 2017, Valtierra-Alvarado 2020 |
| Waist circumference | 4 | 0.207 | –0.07, 0.46 | 0.147 | R | 0.005 | 76 | Christou 2019, Friedrich 2019, Grün 2018, Zaharieva 2017 |
| Fat mass (%) | 3 | 0.311 | 0.08, 0.51 | 0.009 | R | 0.018 | 75 | Friedrich 2019, Grün 2018, Poitou 2011 |
| FBG | 5 | 0.149 | 0.04, 0.25 | 0.006 | R | 0.865 | 0 | Christou 2019, Grün 2018, Poitou 2011, Zaharieva 2017, Valtierra-Alvarado 2020 |
| HbA1c | 5 | 0.199 | –0.002, 0.38 | 0.052 | R | 0.005 | 73 | Christou 2019, Friedrich 2019, Poitou 2011, Zaharieva 2017, Valtierra-Alvarado 2020 |
| Fasting insulin | 3 | 0.227 | 0.11, 0.34 | <0.001 | R | 0.654 | 0 | Christou 2019, Grün 2018, Poitou 2011 |
| HOMA-IR | 3 | 0.236 | 0.12, 0.35 | <0.001 | R | 0.879 | 0 | Christou 2019, Grün 2018, Poitou 2011 |
| TG | 5 | 0.288 | 0.12, 0.44 | 0.001 | R | 0.081 | 52 | Christou 2019, Friedrich 2019, Grün 2018, Zaharieva 2017, Valtierra-Alvarado 2020 |
| TC | 4 | 0.084 | –0.09, 0.25 | 0.343 | R | 0.186 | 38 | Christou 2019, Friedrich 2019, Grün 2018, Zaharieva 2017 |
| LDL-C | 3 | –0.008 | –0.29, 0.27 | 0.957 | R | 0.087 | 59 | Christou 2019, Grün 2018, Zaharieva 2017 |
| HDL-C | 5 | –0.057 | –0.24, 0.13 | 0.557 | R | 0.046 | 59 | Christou 2019, Friedrich 2019, Grün 2018, Zaharieva 2017, Valtierra-Alvarado 2020 |
| VLDL | 2 | 0.074 | –0.15, 0.29 | 0.514 | R | 0.610 | 0 | Zaharieva 2017, Valtierra-Alvarado 2020 |
| CRP | 4 | 0.285 | 0.13, 0.43 | 0.001 | R | 0.096 | 53 | Christou 2019, Friedrich 2019, Grün 2018, Zaharieva 2017 |
| **Non-classical monocytes** | | | | | | | | |
| Age | 3 | 0.109 | –0.01, 0.23 | 0.079 | R | 0.591 | 0 | Christou 2019, Grün 2018, Poitou 2011 |
| BMI | 5 | 0.257 | 0.02, 0.47 | 0.035 | R | 0.002 | 77 | Christou 2019, Grün 2018, Poitou 2011, Zaharieva 2017, Valtierra-Alvarado 2020 |
| Waist circumference | 3 | 0.023 | –0.15, 0.20 | 0.797 | R | 0.940 | 0 | Christou 2019, Grün 2018, Zaharieva 2017 |
| Fat mass (%) | 2 | 0.177 | –0.22, 0.53 | 0.387 | R | 0.017 | 82 | Grün 2018, Poitou 2011 |
| FBG | 5 | 0.192 | –0.03, 0.40 | 0.086 | R | 0.006 | 72 | Christou 2019, Grün 2018, Poitou 2011, Zaharieva 2017, Valtierra-Alvarado 2020 |
| HbA1c | 4 | 0.160 | –0.07, 0.37 | 0.170 | R | 0.021 | 69 | Christou 2019, Poitou 2011, Zaharieva 2017, Valtierra-Alvarado 2020 |
| Fasting insulin | 3 | 0.338 | 0.09, 0.55 | 0.008 | R | 0.027 | 72 | Christou 2019, Grün 2018, Poitou 2011 |
| HOMA-IR | 3 | 0.325 | 0.06, 0.55 | 0.018 | R | 0.015 | 76 | Christou 2019, Grün 2018, Poitou 2011 |
| TG | 5 | 0.102 | –0.04, 0.24 | 0.159 | R | 0.195 | 34 | Christou 2019, Grün 2018, Poitou 2011, Zaharieva 2017, Valtierra-Alvarado 2020 |
| TC | 3 | 0.002 | –0.39, 0.39 | 0.992 | R | 0.007 | 80 | Christou 2019, Grün 2018, Zaharieva 2017 |
| LDL-C | 3 | 0.079 | –0.25, 0.39 | 0.637 | R | 0.039 | 69 | Christou 2019, Grün 2018, Zaharieva 2017 |
| HDL-C | 5 | –0.260 | –0.45, –0.06 | 0.013 | R | 0.011 | 69 | Christou 2019, Grün 2018, Poitou 2011, Zaharieva 2017, Valtierra-Alvarado 2020 |
| VLDL | 2 | 0.109 | –0.12, 0.33 | 0.354 | R | 0.304 | 6 | Zaharieva 2017, Valtierra-Alvarado 2020 |
| CRP | 3 | 0.152 | –0.07, 0.36 | 0.177 | R | 0.103 | 56 | Christou 2019, Poitou 2011, Zaharieva 2017 |

Abbreviations: CRP, C-reactive protein; FBG, fasting blood glucose; HOMA-IR, Homeostatic Model Assessment of Insulin Resistance; TC, total cholesterol; TG, triglycerides; HDL-C; high-density lipoprotein cholesterol; LDL-C, low-density lipoprotein cholesterol; VLDL, very low-density lipoprotein.

# Supplementary Figure


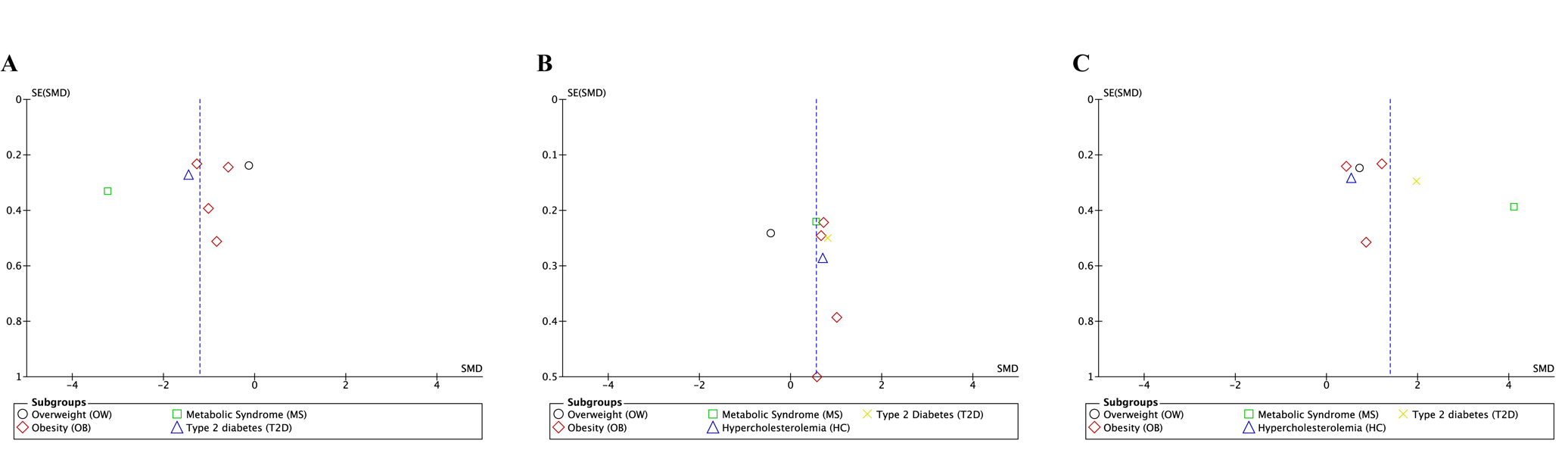


**Supplementary Figure 1.** Funnel plots of meta-analysis evaluating the association between cardiometabolic disorders and the distribution of classical (A), intermediate (B), and nonclassical (C) monocytes.
